# Sensor-Based Technologies for the Detection of Unwanted Loneliness in Older Adults: A Systematic Review

**DOI:** 10.3390/s26072028

**Published:** 2026-03-24

**Authors:** María Mercedes Párraga Vico, Juana María Morcillo Martínez, Juan F. Gaitán-Guerrero, Juan Luis Herreros Bódalo, Macarena Espinilla Estévez, Juan Carlos Cuevas Martínez

**Affiliations:** 1 University of Jaén, 23071 Jaén, Spain; mpvico@ujaen.es; 2 Department of Psychology, University of Jaén, 23071 Jaén, Spain; jmmorcil@ujaen.es; 3 Department of Computer Science, University of Jaén, 23071 Jaén, Spain; jgaitan@ujaen.es; 4 Department of Telecommunication Engineering, University of Jaén, 23700 Linares, Spain; herreros@ujaen.es (J.L.H.B.); jccuevas@ujaen.es (J.C.C.M.)

**Keywords:** loneliness detection, older adults, passive sensing, machine learning, healthcare IoT, multimodal data fusion

## Abstract

**Highlights:**

**What are the main findings?**

- Passive sensor technologies combined with artificial intelligence and multimodal data fusion have great potential for detecting behavioral markers associated with unwanted loneliness and social isolation in older adults.
- Artificial intelligence models based on multimodal fusion achieve greater accuracy than unimodal approaches in predicting states of loneliness and isolation in older adults.

**What are the implications of the main findings?**

- These technologies enable objective assessment, complementing traditional self-reporting tools.
- Their implementation in real-world settings requires addressing challenges of user acceptance and validation in different samples.

**Abstract:**

**Background:** Unwanted loneliness and social isolation in older adults are public health problems with negative effects on physical and mental health. The usual assessment tools, based on self-report questionnaires, have limitations in capturing these phenomena continuously and objectively. **Objective**: We aimed to critically analyze recent scientific evidence on the use of passive sensor technologies combined with artificial intelligence for the detection of unwanted loneliness and social isolation in older adults. **Methods:** Studies were reviewed in databases (PubMed, Scopus, Web of Science, and IEEE Xplore) that used wearable devices, environmental sensors in the home, smartphones, and multimodal fusion approaches. This systematic review was conducted following the PRISMA 2020 guidelines. **Results**: Behavioral variables derived from passive monitoring, such as mobility, time away from home, sleep patterns, and digital interactions, are consistently associated with measures of loneliness and social isolation. Likewise, artificial intelligence models based on the combination of multiple data sources show better predictive performance than unimodal approaches. **Conclusions:** Sensor-based technologies can complement traditional assessment methods, although their practical application requires overcoming challenges related to methodological validation, user acceptance, and ethical considerations.

## 1. Introduction

Unwanted loneliness and social isolation are increasingly recognized as significant public health issues among older adults. Persistent loneliness has been consistently associated with adverse mental health effects, including cognitive decline, depression, sleep disturbance, anxiety, dementia, and even suicidal ideation [1,2,3,4]. In addition, physical health is affected, as there is evidence linking loneliness to cardiovascular disease, hypertension, stroke, frailty, obesity, and functional decline [1,2,3,4,5,6]. These effects are partly mediated by health-related behaviors: socially isolated people tend to be less physically active, have poorer diets, smoke more, and experience greater difficulty quitting smoking, further increasing their risk of disease [6,7].

Large cohort studies and meta-analyses indicate that loneliness, social isolation, or living alone may increase the risk of premature mortality by approximately 26–32% [8,9]. Loneliness is also associated with increased use of healthcare services, including more frequent visits to the doctor and emergency room, especially when combined with social isolation [5,10]. Due to their widespread impact, loneliness and social isolation have been labeled “geriatric giants” by public health authorities, underscoring the need for systematic identification and intervention [11]. Therefore, addressing these issues is a priority not only for individual well-being, but also for health systems and policy planning.

Although self-report scales such as the University of California, Los Angeles (UCLA) Loneliness Scale and the Social and Emotional Loneliness Scale for Adults (SESLA) are widely used and show good internal consistency, recent methodological reviews point to limitations in their validity and ability to adequately capture loneliness [12,13]. Very short forms (1–3 items) are practical for large-scale surveys, but lack sufficient detail for a thorough clinical assessment [12,13,14,15]. In addition, self-reports are subject to social desirability bias and stigma, which may lead older adults to underestimate their feelings of loneliness [16,17]. Validation studies have mainly focused on younger populations with higher education or who use the Internet, limiting their generalizability to older, more frail individuals with cognitive impairment or from culturally diverse backgrounds [12,13,18]. It is important to note that subjective indicators of loneliness and objective indicators of social isolation (e.g., social network size, frequency of social contacts, time spent outside the home) are only modestly correlated, reflecting distinct constructs that should be taken into account in research on aging [19,20]. Traditional questionnaires provide episodic “snapshots” and are influenced by memories and current mood, making them unsuitable for capturing everyday dynamics [17]. Evidence from intelligent sensor systems in homes, known as Ambient Assisted Living (AAL), suggests that continuous, discreet monitoring of behavior—such as activity in the home, time spent outside the home, and computer or cell phone use—can complement self-reports, although sensor-based measurements do not perfectly match questionnaire scores [17]. In summary, validated loneliness questionnaires remain reliable, but in order to continuously and accurately assess social isolation in older adults, they must be complemented by objective indicators and tools adapted to each context.

Advances in sensor technology have opened up new possibilities for the objective monitoring of behaviors associated with social isolation and loneliness in older adults. In general, wearable sensors (activity trackers), smartphones, and environmental sensors can complement self-assessments and clinical assessments by providing objective, continuous measurements of behaviors associated with social isolation, but they do not yet constitute independent, clinically robust detectors [21,22].

The incorporation of Artificial Intelligence (AI) techniques is revolutionizing the monitoring of mental and behavioral health in aging through digital phenotyping and passive detection [23,24]. These approaches enable continuous, real-world assessment. In particular, the fusion of multimodal data from wearables, environmental sensors, and smartphones allows for the construction of more robust predictive models that overcome the limitations of episodic clinical assessments [25,26]. However, recent reviews in the specific field of loneliness highlight that the translation of these systems into clinical practice and care is hampered by critical challenges of methodological standardization, validation in representative samples, interpretability, and ethical considerations [21,27,28].

Despite growing interest in sensor-based approaches, the available evidence remains fragmented, methodologically heterogeneous, and rarely focused specifically on older adults. As a result, the field is shaped by isolated studies that require a review capable of going beyond the mere description of devices and critically analyzing the ability of these technologies to infer experiences of loneliness, the potential of multimodal data fusion, and the ethical and practical barriers to their actual implementation in social and healthcare settings. Therefore, the objective of this review is to comprehensively and critically examine the recent scientific literature on the use of sensor-based and artificial intelligence technologies for the detection of unwanted loneliness and social isolation in older adults.

Throughout this review, we adopt a conceptual and operational distinction between loneliness and social isolation, following established gerontological frameworks [19,20].

Loneliness is defined as a subjective, negative emotional state arising from a perceived discrepancy between desired and actual social relationships. It is typically measured using validated self-report scales such as the UCLA Loneliness Scale (versions 3, 20, or 3-item) or the Social and Emotional Loneliness Scale for Adults (SESLA). Social isolation is defined as an objective, quantifiable state of reduced social network size, infrequent social contacts, or limited participation in social activities. It is operationalized through indicators such as time spent outside the home, frequency of visits, living alone, or low social interaction frequency, often derived from sensor data or behavioral logs.

The manuscript is structured into four main sections. Section 2 describes the materials and methods used to conduct the narrative review. Section 3 presents the results, organized around the main types of sensors, behavioral markers, and analytical approaches used to assess loneliness and social isolation in older adults. Finally, Section 4 discusses these findings in light of previous literature and possible future lines of research.

## 2. Materials and Methods

### 2.1. Study Design

This systematic review is reported in accordance with the Preferred Reporting Items for Systematic Reviews and Meta-Analyses (PRISMA) 2020 statement [29] (see Supplementary Materials). This review was not registered. The objective is to synthesize the existing literature on sensor-based technologies for detecting unwanted loneliness and social isolation in older adults. This methodological choice is justified by the emerging and interdisciplinary nature of the field—which combines engineering, artificial intelligence, and health sciences—and by the considerable methodological heterogeneity of the available studies.

### 2.2. Study Selection Process

The study selection process was conducted in three phases. First, two reviewers (MMPV and JFGG) independently screened titles and abstracts against the eligibility criteria. Disagreements were resolved by consensus or by consulting a third reviewer (MEE). Second, full texts of potentially eligible articles were retrieved and assessed independently by the same two reviewers. Third, references of included studies were manually screened to identify additional relevant records. Duplicates were automatically detected using Zotero 7.0.32 (64-bit) and manually verified.

### 2.3. Search Strategy and Information Sources

The literature search was conducted in the PubMed, Scopus, Web of Science, and IEEE Xplore databases, selected for their complementary coverage of biomedical, technological, and engineering literature. The search focused on articles published between January 2017 and February 2025, a period that coincides with the rise of passive monitoring and smart home approaches applied to aging.

Combinations of keywords and controlled terms related to the following were used: loneliness, social isolation, unwanted loneliness; older adults, elderly, aging population; sensors, wearables, smart home, ambient assisted living, passive sensing; machine learning, artificial intelligence, digital phenotyping. The terms were adapted to the specific syntax of each database, and Boolean operators (AND, OR) were used to combine search concepts. The search was conducted between October 2025 and December 2025. The complete search strings used for each database are provided in Appendix A.

**Figure 1:**
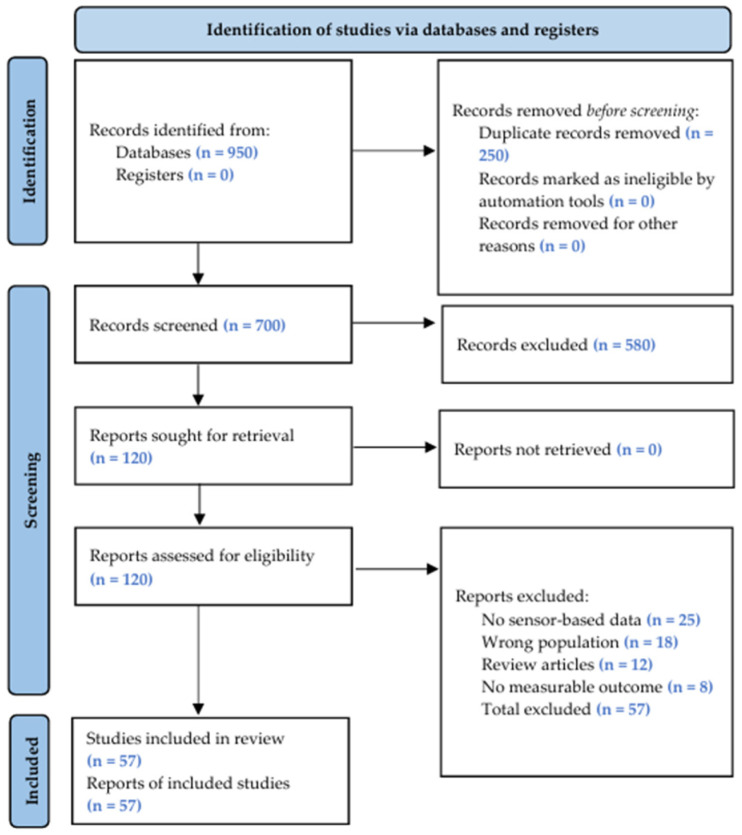
PRISMA 2020 flow diagram of the study selection process.

The flow of the literature search and selection process is summarized in Figure 1, following the PRISMA 2020 guidelines [29]. A total of 950 records were identified, of which 250 were duplicates. After screening 700 titles and abstracts, 580 records were excluded. The full text of 120 reports was assessed for eligibility, of which 63 were excluded (25 no sensor-based data, 18 wrong population, 12 review articles, 8 no measurable outcome). A total of 57 studies met the inclusion criteria and were included in this review.

### 2.4. Data Extraction and Synthesis

From each included study, the following information was extracted: author(s) and year of publication; sample size and population characteristics (e.g., community-dwelling, residential care, clinical condition); study setting (e.g., home, laboratory, population-based); type of sensor technology used (e.g., wearables, environmental sensors, smartphones); behavioral or digital markers extracted from sensor data; target construct (loneliness or social isolation); predictive model or analytical approach; validation method (e.g., cross-validation, hold-out, external validation); and main performance metrics (e.g., accuracy, AUC, correlation coefficients). Data extraction was performed independently by two reviewers (MMPV and JFGG), and discrepancies were resolved through discussion or consultation with a third reviewer (MEE). Due to the heterogeneity of the included studies, a structured narrative synthesis was conducted, organizing findings according to sensor types, behavioral markers, and analytical approaches, as presented in Section 3.

### 2.5. Risk of Bias Assessment

A formal risk of bias assessment using a standardized tool was not conducted due to the heterogeneity of the included studies and the exploratory nature of this review. However, a narrative summary of study limitations (e.g., sample sizes, validation strategies) is provided in Section 3.6.

### 2.6. Eligibility Criteria and Study Selection

The following inclusion criteria were established: (1) studies focusing on older adults (≥60 years); (2) use of passive detection technologies (wearables, environmental sensors, smartphones); (3) objective of detecting, predicting, or correlating with loneliness or social isolation; (4) presentation of analyzable quantitative or qualitative results on the validity or usefulness of the technology; (5) studies based solely on self-reports, systematic reviews, opinion articles, studies without validation results, and those whose main focus was not loneliness/isolation were excluded. The identified articles were organized and managed using Zotero software. Table 1 presents the detailed eligibility criteria.

## 3. Results

**Figure 2:**
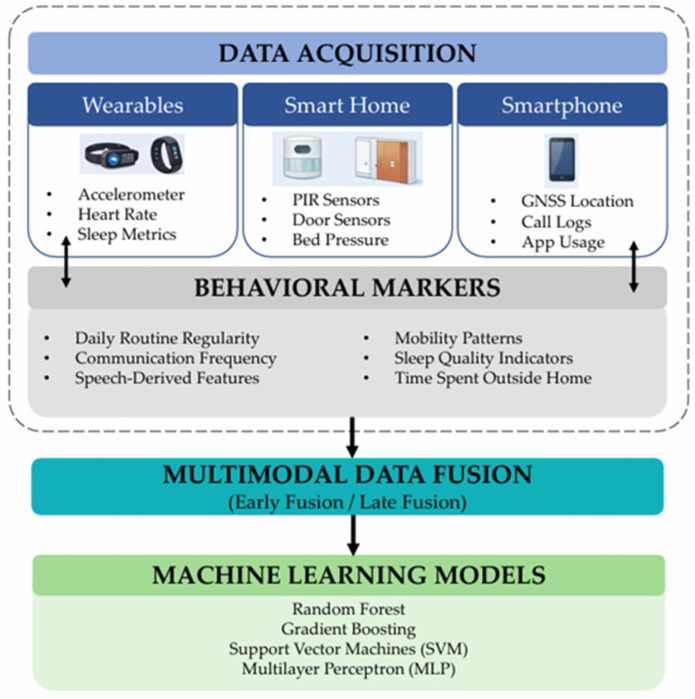
Technological flow for the detection of loneliness and social isolation in older adults using sensors.

The reviewed evidence on sensor-based technologies for the detection of loneliness and social isolation in older adults is structured around the technological workflow illustrated in Figure 2. This framework integrates three key stages: data acquisition from multiple sensor platforms (wearables, smart home devices, and smartphones), the extraction of behavioral markers associated with loneliness, and analytical approaches based on multimodal data fusion and machine learning models.

### 3.1. Sensors and Monitoring Platforms

Wearable devices and smartphones can track physical activity, location, phone and app usage, heart rate, and sleep patterns [21,30], while smart home environmental sensors, such as motion detectors, door contacts, bed and mattress sensors, temperature and humidity monitors, and appliance usage, can provide additional behavioral context [27,31]. New smart textiles integrated into clothing or furniture aim to provide comfortable and continuous monitoring [22,31]. The reviewed literature describes a growing use of wearable devices, environmental sensors, and mobile platforms for monitoring behavioral and physiological variables related to social isolation and loneliness in older adults [17,21,27,28,30,32,33,34]. Table 2 below presents the main types of sensors used, the variables they measure, and examples of their application, according to recent scientific literature.

To complement the descriptive information presented in Table 2, Table 3 provides a critical assessment of the strengths and limitations of each sensor category, emphasizing their applicability and methodological constraints in loneliness and social isolation detection.

The comparative analysis highlights that passive sensing technologies offer high ecological validity but limited interpretability, whereas wearable and smartphone-based approaches provide richer multimodal data at the expense of usability and privacy concerns. Audio–video and physiological sensing approaches remain promising but are still constrained by ethical, technical, and deployment challenges. Future research should prioritize multimodal sensor fusion, explainable AI models, and longitudinal validation in real-world environments to improve robustness and generalizability.

### 3.2. Sensor-Derived Behavioral Markers and Predictive Models for Loneliness and Social Isolation

Based on the variables measured by the sensors described in Section 3.1, the literature identifies various behavioral markers associated with loneliness and social isolation, including time spent outside the home, room location patterns, daytime naps, reduced mobility, and sleep disturbances, which have been integrated into predictive models [54,55]. Similarly, variables related to telephone and computer use, as well as the frequency of social visits, have been incorporated into different modeling approaches [56]. The studies reviewed report performance metrics ranging from moderate to high values, depending on the type of sensor, the behavioral marker analyzed, and the model used. For example, systems based on PIR sensors and door contacts in smart homes have shown correlations with loneliness scores on the UCLA Scale (r ≈ 0.48), while multisensory platforms have achieved R^2^ values of ≈ 0.86 using features derived from bed sensors and environmental parameters [35,55]. It should be noted, however, that most of these high-performance metrics have been achieved in controlled or semi-controlled research settings with small samples, and their generalizability to real-world conditions remains to be demonstrated.

Notably, certain sensor-derived markers are more conceptually aligned with objective social isolation than with subjective loneliness. For instance, time spent outside the home, frequency of outings, and number of visits detected via door sensors or proximity beacons directly quantify social contact opportunities [17,33]. In contrast, markers such as sleep fragmentation, mobility variability, or linguistic traits in speech have been associated with the subjective experience of loneliness, although the mechanisms linking them remain less understood and require further validation [39,57,58].

To reflect the conceptual distinction between subjective loneliness and objective social isolation established in Section 1, the reviewed studies are organized into two tables. Table 4 includes studies predicting loneliness using validated self-report scales (e.g., UCLA, SESLA). Table 5 compiles studies focusing on objective social isolation, operationalized through sensor-derived behavioral proxies such as mobility patterns, home exits, or living alone. This separation avoids ambiguity in interpreting the evidence, as both constructs, although related, are measured differently.

### 3.3. Multimodal Data Fusion and Artificial Intelligence Approaches

Combining data from different types of sensors using machine learning techniques allows for the construction of more robust predictive models for detecting loneliness. Mobile and wearable sensors, including accelerometers, heart rate monitors, sleep trackers, GNSS (Global Navigation Satellite System) geolocation, and smartphone usage logs, are widely used to infer mood, stress, depression, anxiety, and daily functioning through passive and personal detection approaches [25,62,63]. In controlled or semi-controlled environments, stress and affect classification based on multimodal biological signals has achieved high accuracy, often exceeding 90% [25,64].

The fusion of heterogeneous sensor modalities—including wearables, environmental sensors, and smartphones—has been suggested as a strategy to improve predictive robustness compared to single-sensor approaches, supporting the concept of a ‘behavior’ composed of multiple digital markers [26,65,66]. Multimodal fusion techniques have been applied to activity recognition, cardiovascular risk estimation, and the prediction of cognitive and mobility outcomes in older adults, with ensemble and gradient boosting models showing moderate to high correlations with clinical reference assessments in research settings [26,65,67].

**Figure 3:**
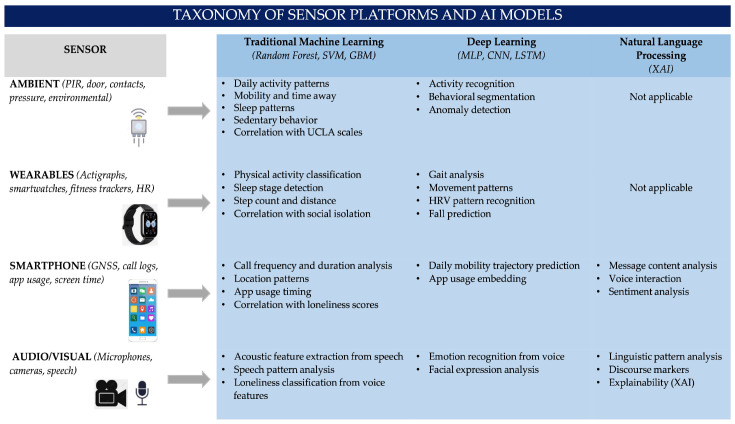
Taxonomic overview of sensor platforms and AI models.

Figure 3 presents a taxonomic overview of the sensor-based AI landscape identified in this review. The diagram organizes four sensor categories (ambient, wearables, smartphone, audio/visual) against three AI model families (Traditional ML, Deep Learning, NLP), with representative applications derived from the 57 included studies.

The reviewed studies employ diverse AI approaches that can be categorized into three main families:

- Traditional machine learning: Algorithms such as Random Forest, Gradient Boosting, and Support Vector Machines are most commonly used due to their interpretability and good performance with tabular data extracted from sensors [39,57,59,60]. These models require manual feature engineering but offer better transparency for clinical applications [58].
- Deep learning: Although less frequent, deep learning approaches including multilayer perceptrons and convolutional neural networks have been applied to raw sensor data, particularly for activity recognition and speech analysis [58,61]. These methods can automatically learn features but require larger datasets and raise concerns about interpretability (‘black box’ issue).
- Natural Language Processing: Specific to audio-based sensing, NLP techniques including explainable AI (XAI) have been used to analyze linguistic traits in interviews and speech patterns associated with loneliness [57,58]. These approaches offer unique insights into subjective experiences but require careful validation across languages and cultures.

However, no large-scale systematic studies have yet directly compared unimodal versus multimodal approaches specifically for loneliness detection in older adults. The current evidence, while promising, remains preliminary and derives primarily from controlled or semi-controlled environments with limited sample sizes and internal validation only.

### 3.4. Digital Phenotyping Applications in Older Adult Populations

In the included studies, digital phenotyping has been applied primarily for the continuous assessment of physical activity, mobility, sleep, cognition, and mood in older adults, using wearable devices, smartphones, and home-based detection systems [67,68,69]. Several studies have demonstrated the feasibility and acceptability of long-term monitoring using GNSS geolocation and wearable devices in older adults living in the community, including those with mild cognitive impairment (MCI) or early-stage dementia [69,70].

The mobility and living space characteristics derived from sensor data show consistent associations with cognitive function, physical performance, and depressive symptoms [70,71]; variables closely related to social isolation and psychosocial vulnerability in older adults. Similarly, sleep metrics such as fragmentation and efficiency, obtained using portable devices, have been associated with daily fluctuations in depressive symptoms in socially vulnerable older adults [23].

In addition, multisensory home systems combined with artificial intelligence techniques have demonstrated their potential to distinguish between normal aging, MCI, and early dementia, achieving high classification performance in research settings [26,67]. Table 6 shows the evidence on digital phenotyping in older adults, displaying the dimensions of aging, sensors used, derived variables, and relevant results.

### 3.5. Inferring Social and Contextual Behavior from Sensor Data

Beyond individual health metrics, AI-based analysis of sensor data is increasingly being used to infer social and contextual behavior. Computer vision, smartphone sensors, and wearable device data enable the quantification of social interactions, sociability, and living space, including community engagement derived from GNSS, phone communication patterns, and environmental sensing [55,62,63]. Although both classical and deep learning methods can recognize interaction patterns, daily routines, and behavioral changes, their generalization and clinical reliability remain limited by small sample sizes, homogeneous cohorts, and heterogeneous methodological choices across studies [24,63,64].

### 3.6. Summary of Evidence Levels

Based on the reviewed studies, the evidence can be categorized into three levels according to methodological rigor, sample size, and validation strategies.

Relatively robust evidence: Associations between basic behavioral markers (time outside home, mobility patterns, sleep fragmentation) and loneliness or social isolation are supported by multiple studies with consistent findings across different populations and settings [17,33,35,39]. These markers have been validated using standardized instruments (e.g., UCLA Loneliness Scale) and show moderate correlations in community-dwelling older adults [17,35].

Promising but preliminary evidence: Multimodal data fusion approaches demonstrate potential for improving predictive performance, with some studies reporting AUC values > 0.85 [39,80]. However, direct comparisons with unimodal approaches are lacking, and most studies are limited by small sample sizes (typically n < 100), lack of external validation, and controlled settings [25,26,65].

Exploratory evidence: Emerging approaches including linguistic analysis using natural language processing [57,58] speech pattern recognition [59] and digital phenotyping for cognitive impairment [67,69] represent innovative methodologies requiring replication in larger, diverse populations before clinical translation.

Direct performance comparisons between studies are not feasible due to substantial heterogeneity in dataset characteristics, evaluation metrics, problem formulations, and validation strategies. This heterogeneity underscores the need for standardized benchmarks and reporting guidelines [21,27,28].

## 4. Discussion

Based on the findings presented, the discussion is organized into five subsections: (1) summary of the technological and methodological findings identified; (2) the contribution of sensors and AI to the detection of loneliness; (3) the advantages and limitations of passive monitoring; (4) ethical considerations, privacy, and acceptance by users; and (5) future lines of research.

### 4.1. Synthesis of Principal Findings

This narrative review has identified and organized recent evidence on the use of sensor-based technologies and artificial intelligence (AI) for the detection of unwanted loneliness and social isolation in older adults. The results indicate that there are three main technological components that interact in a continuous flow: (a) sensor platforms (wearables, environmental, and smartphone) that collect raw data; (b) behavioral and digital markers derived from that data (mobility, sleep, social interaction, patterns at home, linguistic traits); and (c) machine learning (ML) and multimodal fusion analytical approaches that transform the markers into predictions or correlations with validated loneliness scales [17,39,53]. The literature converges in indicating that, although self-reports remain essential, passive sensors offer objective, continuous, and contextual measurement of behaviors associated with loneliness, overcoming limitations such as social desirability bias or the episodic nature of questionnaires [21,27]. However, the field is still in an emerging phase, with studies presenting considerable methodological heterogeneity, small sample sizes, and limited longitudinal validation [37,81].

### 4.2. The Role of Sensor Technologies and AI in Loneliness Detection

Passive sensors enable the capture of rich, multidimensional digital phenotypes of aging. The review shows that markers such as reduced time spent outside the home, low morning mobility, sleep fragmentation, and decreased frequency of telephone communications show consistent, albeit moderate, associations with loneliness scores [35,39,59]. Multimodal data fusion (e.g., combining data from wearables, environmental sensors, and smartphones) is emerging as a key strategy for improving the robustness and predictive performance of models, overcoming the limitations of unimodal approaches [25,26].

Machine learning algorithms, particularly ensemble models such as Random Forest and Gradient Boosting, have shown promise in modeling the complex relationship between these digital markers and loneliness/isolation constructs, achieving performance metrics such as AUC > 0.85 in some studies [39,80]. However, most studies have been conducted in controlled or semi-controlled environments, and the interpretability of the models (‘black box’ issue) remains a challenge for their clinical acceptance [58].

### 4.3. Advantages and Current Limitations of Passive Sensing

Passive monitoring using sensors offers distinct advantages for assessing loneliness in older adults. Its main strength lies in its ability to capture real-world behavioral dynamics objectively, continuously, and discreetly [64,68]. This is particularly valuable for populations with difficulties in frequent self-reporting or with cognitive impairment. Smart home systems (AAL), for example, have proven useful for inferring patterns of isolation from metrics such as intra-domestic mobility and use of spaces [17,45]. The reviewed evidence indicates that these systems can identify patterns of behavior—such as reduced mobility, use of domestic space, or decreased outings—that show significant associations with standardized loneliness scales, pointing to their potential usefulness as objective indicators [21,27]. The fusion of multimodal data (wearables, environmental, smartphones) is emerging as a key strategy for improving predictive robustness compared to unimodal approaches [26].

However, despite these promising results, the field is still in its early stages, characterized by small sample sizes, heterogeneous methods, inconsistent definitions of loneliness versus social isolation, and limited longitudinal validation [32,37,81]. The strength of this evidence is still limited. Most studies are based on small samples, short follow-up periods, and lack population diversity [21,27]. There is a notable lack of longitudinal studies with consistent methodologies in the field of aging [21].

In addition, privacy, data security, and acceptability, particularly with regard to camera monitoring, are issues of great concern; older people tend to respond positively, but are cautious about potential misuse and lack of human interaction [32,81]. This position is a conceptual discrepancy between objective markers of isolation (e.g., time spent at home) and the subjective experience of loneliness, which do not always correlate [81]. This divergence underscores that sensor technologies should be viewed as a valuable complement to, rather than a replacement for, traditional psychosocial assessments, requiring careful integration of both approaches for a holistic assessment of social well-being in older adults.

From an implementation perspective, the technologies reviewed exhibit varying levels of practical readiness. To provide a more structured perspective on their maturity, it is useful to consider the Technology Readiness Levels (TRL) scale, a standardized framework widely used to assess how mature a technology is before it can be integrated into systems or deployed in real-world settings. Applying this scale to the reviewed technologies:

TRL 7–9 (Mature systems, ready for deployment): Basic sensors (PIR, door contacts, pressure mats) have reached TRL 9, with demonstrated commercial maturity and deployment in real-world pilot studies [17,44]. Commercial wearables (actigraphy, activity trackers) are at TRL 8–9, widely available and validated for activity and sleep monitoring in general populations [37,38,40].

TRL 4–6 (Systems in validation phase): Integrated multimodal systems for loneliness detection (combining wearables + environmental sensors + smartphones) are at TRL 4–5, with validation in controlled or semi-controlled environments, but require demonstration in real-world conditions at scale and overcoming integration challenges [26,44,67].

TRL 1–3 (Proof of concept): Audio and video analysis using NLP for loneliness detection, as well as advanced physiological sensors (EEG, EDA) applied to this specific domain, are at TRL 2–3, currently limited to laboratory settings with small samples and controlled conditions [51,52,57,58]. Smart textiles for loneliness monitoring are in early development stages (TRL 2–3) [22,47].

Considering these challenges, a staged implementation pathway is proposed. In the short term (1–3 years), simple single-modal approaches using commercial sensors are ready for broader deployment [17,37,43]. In the medium term (3–5 years), integrated multimodal systems with machine learning can be piloted [26,44,58]. In the long term (>5 years), advanced AI-driven approaches require further development before clinical adoption [57,61,67].

### 4.4. Ethical, Privacy, and User Acceptance Considerations

The qualitative and review studies included describe concerns related to privacy, autonomy, and user acceptance. The main concerns and conditions for the acceptance of passive monitoring technologies in older adults are summarized in Table 7.

These findings underscore that any implementation of passive sensors must prioritize trust in the system, privacy, and user control. Trust is at the core of acceptability. The design must incorporate an ethical framework based on transparency and a person-centered approach, as these technologies can be perceived as surveillance, eliminating the autonomy they are intended to protect. The adoption of edge computing architectures in IoT environments can be a solution to strengthen privacy. The processing of more sensitive data is carried out in the nodes or sensors themselves, without that information leaving the device. The results that are transmitted are anonymized, reducing the risk of exposure of personal data and reinforcing user trust and security.

Notably, audio and video sensors—despite their rich informational value—are consistently perceived by older adults as more intrusive than ambient environmental sensors. This aligns with the design preferences summarized in Table 7, where non-invasive and discreet solutions are prioritized over camera-based monitoring [82,88,90,91].

The adoption of edge computing architectures in IoT environments can be a solution to strengthen privacy. From a technical perspective, edge computing can be implemented so that raw signals (e.g., accelerometer data, passive infrared motion events) are processed locally on the sensor node. Only aggregated, anonymized features—such as hourly activity counts or time spent outside the home—are transmitted to external servers. Complementary privacy-preserving strategies, such as federated learning, enable model training across distributed devices without centralizing sensitive data.

However, it is important to acknowledge that anonymization techniques (including aggregation, pseudonymization, and differential privacy) are not infallible; residual risks such as model inversion attacks or metadata leakage persist and must be addressed through continuous technical and procedural safeguards.

### 4.5. Future Research Directions

To advance the field from promising prototypes to impactful real-world applications, future research must move beyond technical validation towards a structured, multi-dimensional agenda. Based on the gaps identified in this review, we organize the key priorities into five interconnected research directions, summarized in Table 8. This framework distinguishes between short-term goals (1–3 years), which are immediately actionable, and long-term objectives (>5 years), which require sustained, multi-stakeholder effort.

Crucially, an overarching priority that must permeate all the above directions is equity. Future research must proactively ensure that these technologies are accessible and valid across diverse populations, including people with cognitive impairment, low digital literacy, ethnic minorities, and those living in socioeconomically disadvantaged or rural settings with limited access to technological infrastructure. Without this focus, there is a significant risk of exacerbating existing health disparities in both loneliness and social isolation.

### 4.6. Limitations of This Review

This review has several limitations. First, although we followed PRISMA guidelines and conducted a systematic search, the review was not registered in a database such as PROSPERO. Second, the substantial heterogeneity across studies—in terms of design, sample size, sensors used, and outcome measures—prevented us from conducting a meta-analysis and limited our synthesis to a narrative approach. Third, the exclusion of non-English publications and grey literature may have introduced language or publication bias. Fourth, the overall quality of the included studies remains limited, with most relying on small samples and lacking external validation. Finally, the rapid evolution of technology means that recent innovations may not be fully captured, despite our focus on the 2017–2025 period.

## Figures and Tables

**Table 1:** Eligibility criteria.

Criterion	Inclusion Criteria	Exclusion Criteria
Population	Older adults (≥60 years)	Studies focused on younger adults, caregivers, or the general population without age-specific analysis
Technology	Passive sensing technologies (e.g., wearables, environmental sensors, smartphones)	Active sensing requiring user interaction, questionnaire-only studies, or non-sensor-based methods
Outcome	Detection, prediction, or correlation with loneliness or social isolation	Studies not reporting loneliness or social isolation as an outcome
Study type	Primary research reporting quantitative or qualitative results on validity, feasibility, accuracy, or usefulness	Systematic reviews, opinion articles, editorials, conference abstracts, or studies without validation results
Language	Publications in English	Non-English publications
Publication date	January 2017–February 2025	Publications before January 2017

**Table 2:** Types of Sensors used to Assess Social Isolation and Loneliness in Older Adults.

Sensor Type/Platform	Main Measured Variables	Variables Related to Loneliness/Isolation	References
Motion sensors (PIR, infrared)	Mobility, presence in rooms, daily activity	Time spent in each room, activity/inactivity patterns, mobility	[17,21,27,28,30,32,33,34]
Door contact sensors	Home entrances/exits, room usage	Frequency of outings, time spent outside the home, interior door usage	[17,21,27,30,32,33,34]
Pressure sensors (bed/chair, smart mattress)	Bed/chair presence, sleep parameters	Bedtime, naps, sleep efficiency, sedentary time	[28,32,33,35,36]
Environmental sensors (light, electricity, water, temperature, humidity, air quality)	Appliance usage, thermal comfort, home routines	TV hours, kitchen/bathroom use, shower events, heating patterns	[17,21,27,30,32,33,34,35]
Actigraphs/portable accelerometers	Physical activity, movement patterns	Daily activity level, sedentary time, movement changes	[28,32,37,38,39,40]
Smartwatches and fitness trackers	Activity, sleep, vital signs	Daily steps, sleep, heart rate, physiological variability	[28,31,32,36,37,38,39,41,42]
Smartphone (sensors and usage logs)	Communication, mobility, app usage	Number/duration of calls, messages, social app usage, GNSS	[28,32,37,38,43,44,45]
Proximity sensors (BLE, RFID, tags)	Proximity to objects or people	Being at home vs. away, movement within the home, social encounters	[32,46]
Specific physiological sensors	Biological indicators (HR, temperature, EDA, EEG, ECG)	Heart rate, conductance, temperature, stress associated with loneliness	[28,32,37,38,47,48,49]
Smart textile sensors (clothing/furniture)	Body activity, posture, comfort	Movement, posture, social interaction, continuous monitoring	[22,32,38,47,50]
Audio and video sensors (NLP, cameras)	Verbal interactions, facial expression, language	Voice analysis, speech patterns, non-verbal expressions	[51,52,53]

**Table 3:** Strengths and Limitations of Main Sensor Technologies for Detecting Loneliness and Social Isolation in Older Adults.

Sensor Category	Strengths	Limitations
Motion Sensors (PIR, Infrared)	Fully passive and unobtrusive; enable long-term monitoring; no user burden; capture indoor movement dynamics	Limited to indoor spaces; cannot identify individuals; limited sensitivity to subtle behavioral changes
Door Contact Sensors	Simple and reliable; generate clear binary event data; detect home exits and entries	Only capture door events; miss detailed activity outside the home; limited insight into social interactions
Pressure Sensors (Bed/Chair Sensors)	Passive monitoring of sleep and rest; no wearable required; capture nocturnal behavior	Restricted to specific furniture; cannot detect sleep stages; performance affected by multiple occupants
Environmental Sensors (Multi-Sensor Home Systems)	Capture contextual home activity; support long-term deployment; provide behavioral context for ADLs	Provide indirect measures requiring interpretation; sensitive to environmental changes; installation infrastructure required
Wearable Sensors	Continuous physiological and activity monitoring; high temporal resolution; capture indoor and outdoor movement; commercially available	Require charging and maintenance; adherence may decrease; potential discomfort or abandonment
Smartphone-Based Sensing	Leverages existing personal devices; captures communication and mobility data; supports multimodal analytics	Privacy concerns; battery consumption; digital divide among older adults; platform heterogeneity
Physiological Sensors (ECG, EDA, EEG)	Capture objective stress-related biomarkers; high measurement precision; potential emotional correlates	Require contact-based setup; typically limited to controlled or laboratory environments; complex signal processing
Audio and Video Sensors	Direct assessment of social interaction; enable linguistic, paralinguistic, and behavioral analysis; rich contextual information	Highly intrusive; strong privacy and ethical concerns; computationally intensive; language-dependent

**Table 4:** Studies Predicting Subjective Loneliness in Older Adults Using Sensors and Predictive Models.

Instrument/Scale	Sensor/Features	Model	n	Population	Setting	Validation	Metric	References
UCLA 3-item	PIR, door contacts, PC/phone use	Multiple linear regression	30	Community, homes	Home	Hold-out	R^2^ = 0.35	[17]
EMA + validated scales	Sleep, physical activity, health, EMA	Gradient Boosting	78	Community, predementia	Community	Cross-validation	AUC = 0.887	[39]
UCLA (4 factors)	Call logs, GPS location	Multiple classifiers	52	Community	Community	Not specified	Accuracy, sensitivity, specificity by factor	[59]
UCLA	Linguistic traits (interviews)	Explainable AI (XAI)	84	Older adults	Laboratory	Hold-out	Accuracy = 0.889; AUC = 0.80; F1 = 0.80	[58]
UCLA (qual + quant)	Linguistic traits (interviews)	ML models	104	Community	Laboratory	Cross-validation	Precision = 94%/76%; Sensitivity = 0.90/0.57; Specificity = 1.00/0.899	[57]
Loneliness scale	Sociodemographic, functional health	Gradient Boosted Trees	4621	Population-based, China	Population-based	Cross-validation	AUC = 0.84	[60]
Loneliness scale	Psychosocial predictors, health	MLP vs. Logistic Regression	1541	Population-based, Spain	Population-based	Not specified	Accuracy = 92.3%; R^2^ Nagelkerke = 0.396	[61]
UCLA	Speech analysis	SVM, Random Forest	96	Community	Laboratory	Cross-validation	Accuracy = 76.5%	[59]

**Table 5:** Studies Predicting Objective Social Isolation in Older Adults Using Sensors and Predictive Models.

Instrument/Scale	Sensor/Features	Model	n	Population	Setting	Validation	Metric	References
EMA (ecological momentary assessment)	Actigraphy (daily physical activity)	Random Forest	78	Community, predementia	Community	10-fold CV	AUC = 0.935; Accuracy = 0.849; F1 = 0.824	[39]
Not applicable (descriptive)	Multimodal: wearables, home sensors	Descriptive	20	Community, post-fracture	Home	Not applicable	Feasibility outcomes	[44]
Not applicable (descriptive)	PIR sensors, door contacts	Descriptive analysis	60	Community, COVID-19	Home	Not applicable	Behavioral changes	[45]
Experimental task	Non-verbal signals (avatar)	ML models	40	Older adults	Laboratory	Cross-validation	To be determined	[51]
Functional decline scales	Multimodal: wearables, sensors	Correlation analysis	15	Community, post-fracture	Home	Not applicable	Preliminary correlations	[36]

**Table 6:** Dimensions, Sensors, Variables, and Findings in Digital Phenotyping of Aging.

Aging Dimension	Sensors/Systems	Extracted Variables	Relevant Findings	References
Physical activity and mobility/life-space	Smartphone (GNSS, accelerometer), wrist wearables, ECG patches with accelerometer, home motion sensors	Time at home, distance traveled, radius of gyration, number of significant locations, circadian routine, PA intensity, steps/day, temporal PA patterns	Higher activity and spatial diversity are associated with better cognition, less functional decline, less depression, and greater community engagement; low PA is linked to higher risk of MCI/dementia and worse executive function	[64,68,72,73]
Cognition and dementia risk	Smartphone (GNSS, app usage, keystrokes), wearables, multisensory home systems (PIR, doors, bed, medication, beacons)	Mobility phenotypes, regularity of habits, typing speed and variability, time of first/last phone interaction, high-resolution PA metrics	Combinations of digital traits (mobility, PA, device usage, home patterns) discriminate between normal aging and early MCI/dementia with good ML model performance	[64,71,72,73]
Mood/depressive symptoms	Smartphone (GNSS, calls/app usage), activity and sleep wearables, bed sensors	Sleep fragmentation and efficiency, activity variability, daily mobility, volume and temporal pattern of calls/screen usage	Lower mobility, more irregular sleep, and certain phone usage patterns are associated with greater severity and variability of depressive symptoms over time	[64,74,75,76,77]
Sleep	Wearables (actigraphy, fitness bands), bed sensors, ECG patches	Sleep duration, nocturnal awakenings, efficiency, night-to-night variability, circadian activity rhythms	Sleep metrics are related to daily mood fluctuations and variability of depressive symptoms; some sleep traits contribute to prediction models of cognitive decline and social isolation	[44,71,74,75,76]
Social isolation and community life	Home motion and door sensors, proximity beacons, smartphone (GNSS, communication logs)	Time away from home, frequency of outings, room presence patterns, call frequency/duration, sociability indicators	Patterns of lower community mobility, fewer visits to places, and less social interaction are associated with social isolation, worse functional status, and greater psychosocial vulnerability	[44,64,67,78,79]

**Table 7:** Main Ethical and Privacy Concerns and Conditions for Acceptance of Passive Monitoring Technologies in Older Adults.

Topic	Key Findings	References
Privacy and data misuse	Older adults often fear misuse by third parties, surveillance, and data leaks.	[32,81,82,83,84,85,86]
Ethics of emotional monitoring	Skepticism about sensors’ ability to “read” emotions like loneliness; concerns about stigma or misinterpretation.	[21,27,32,81]
Conditional acceptance	Many value potential benefits (safety, loneliness detection, aging in place) but only accept systems that are discreet, transparent, and controllable.	[32,81,84,85,87,88]
Autonomy and control	Passive monitoring may threaten perceived autonomy; residents may resist or stop using systems that interfere with their routine or values.	[89,90]
Design preferences	Non-invasive/ambient sensors preferred over video, integrated into familiar objects, with customizable alerts and clear data-sharing rules.	[82,88,90,91]
Trust and aesthetics	Trust in information handling and system reliability, along with non-stigmatizing and aesthetically pleasing design, promote acceptance.	[50,85,88]

**Table 8:** Structured Research Agenda for Sensor-Based Detection of Loneliness and Social Isolation in Older Adults.

Research Direction	Key Priorities and Short-Term vs. Long-Term Goals
Longitudinal and Diverse Cohorts	Short-term: Conduct multi-site studies with larger, more diverse samples (including rural, low-income, and ethnic minority populations). Long-term: Establish long-term cohorts (>5 years) to assess the stability, predictive validity, and causal relationships of digital behavioral markers with loneliness and health outcomes.
Standardization and Benchmarking	Short-term: Develop community-agreed reporting standards for sensor-based loneliness studies (sample, sensors, features, models, validation). Long-term: Create open-source benchmark datasets to allow direct, fair comparison between unimodal and multimodal approaches, and between different AI models.
Implementation Science and Real-World Integration	Short-term: Conduct pragmatic trials to evaluate the integration of simple sensor systems into existing social care workflows. Assess cost-effectiveness, user burden, and technical reliability. Long-term: Develop interoperable platforms that can feed data into electronic health records and trigger timely, ethical interventions.
Ethics, Privacy, and User-Centered Design	Short-term: Operationalize concepts like “functional privacy” and “perceived invasiveness” through co-design studies with older adults, caregivers, and practitioners. Long-term: Develop and validate privacy-preserving technologies (e.g., edge computing, federated learning) that are transparent and give users meaningful control over their data.
Clinical Translation and Risk Management	Short-term: Quantify the clinical risks of false positives (unnecessary anxiety, intervention) and false negatives (missed support) in pilot implementation studies. Long-term: Establish clear clinical guidelines on how to interpret and act upon alerts generated by these systems, ensuring they augment, not replace, human care.

## Data Availability

No new data were created or analyzed in this study. Data sharing is not applicable to this article.

## Supplementary Materials

The following supporting information can be downloaded at: https://www.mdpi.com/article/10.3390/s26072028/s1, Table S1: PRISMA 2020 for Abstracts Checklist; Table S2: PRISMA 2020 Checklist.

## Abbreviations

The following abbreviations are used in this manuscript:

AAL	Ambient Assisted Living
AI	Artificial Intelligence
AUC	Area Under the Curve
BLE	Bluetooth Low Energy
ECG	Electrocardiogram
EDA	Electrodermal Activity
EEG	Electroencephalography
EMA	Ecological Momentary Assessment
GBM	Gradient Boosting Machine
GNSS	Global Navigation Satellite System
HR	Heart Rate
HRV	Heart Rate Variability
IoT	Internet of Things
ML	Machine Learning
MLP	Multilayer Perceptron
MCI	Mild Cognitive Impairment
NLP	Natural Language Processing
NMAE	Normalized Mean Absolute Error
NRMSE	Normalized Root Mean Square Error
PA	Physical Activity
PIR	Passive Infrared
PRISMA	Preferred Reporting Items for Systematic Reviews and Meta-Analyses
RF	Random Forest
RFID	Radio-Frequency Identification
SESLA	Social and Emotional Loneliness Scale for Adults
UCLA	University of California, Los Ángeles Loneliness Scale
XAI	Explainable Artificial Intelligence

## Appendix A

Table A1 presents the complete search strings used for each database, following the PRISMA 2020 guidelines [29]. The searches were conducted between October and December 2025, as specified in Section 2.3.

**Table A1:** Search strings for each database.

Database	Search String
PubMed	(loneliness OR “social isolation” OR “social behavior” OR “social interaction”) AND (“older adults” OR elderly OR aging OR aged) AND (sensor* OR wearable* OR “smart home” OR “ambient assisted living” OR “passive sensing” OR monitoring)
Scopus	TITLE-ABS-KEY ((loneliness OR “social isolation”) AND (“older adults” OR elderly) AND (sensor* OR wearable* OR “smart home”))
Web of Science	TS = (loneliness OR “social isolation”) AND TS = (“older adults” OR elderly) AND TS = (sensor* OR wearable* OR “smart home”)
IEE Xplore	(“All Metadata”:loneliness OR “All Metadata”:”social isolation”) AND (“All Metadata”:”older adults” OR “All Metadata”:elderly) AND (“All Metadata”:sensor* OR “All Metadata”:wearable*)
